# Choline and betaine intake and risk of breast cancer among post-menopausal women

**DOI:** 10.1038/sj.bjc.6605510

**Published:** 2010-01-05

**Authors:** E Cho, M D Holmes, S E Hankinson, W C Willett

**Affiliations:** 1 Channing Laboratory, Department of Medicine, Harvard Medical School and Brigham and Women's Hospital, Boston, MA, USA; 2 Department of Nutrition, Harvard School of Public Health, Boston, MA, USA; 3 Department of Epidemiology, Harvard School of Public Health, Boston, MA, USA

**Keywords:** choline, betaine, breast cancer, women, cohort

## Abstract

**Background:**

Choline and betaine, similar to folate, are nutrients involved in one-carbon metabolism and hypothesised to reduce breast cancer risk. No prospective study among post-menopausal women has examined choline and betaine intakes in relation to breast cancer risk.

**Methods:**

We examined the intake of choline and betaine and breast cancer risk among 74 584 post-menopausal women in the Nurses’ Health Study. Nutrient intake was assessed using a validated food-frequency questionnaire six times since 1984. During 20 years of follow-up from 1984 until 2004, we documented 3990 incident cases of invasive breast cancer.

**Results:**

Overall, choline (mean±s.d.; 326±61 mg per day) and betaine (104±33 mg per day) intake was not associated with a reduced risk of post-menopausal breast cancer. Participants in the highest quintile of intakes had multivariate relative risks of 1.10 (95% confidence interval (95% CI): 0.99–1.22; *P*-value, test for trend=0.14) for choline and 0.98 (95% CI: 0.89–1.09; *P*-value, test for trend=0.96) for betaine, compared with those in the lowest quintiles of intakes. The results were similar in breast cancer stratified by hormone receptor (oestrogen receptor/progesterone receptor) status. The association between choline intake and breast cancer risk did not differ appreciably by alcohol intake (non-drinker, <15 or 15+ g per day) or several other breast cancer risk factors, including family history of breast cancer, history of benign breast disease, body mass index, post-menopausal hormone use, and folate intake.

**Conclusion:**

We found no evidence that higher intakes of choline and betaine reduce risk of breast cancer among post-menopausal women.

Choline and betaine are nutrients involved in one-carbon metabolism, a network of biochemical reactions that transfer methyl groups from one compound to another (Mason, 2003). One-carbon metabolism is involved in the methylation of DNA and RNA and subsequently influences gene stability and expression. One-carbon metabolism also mediates nucleotide synthesis; thus, perturbation of the metabolism may lead to chromosomal breaks and disruption of DNA repair. Choline can convert to betaine, which can donate the methyl group to homocysteine as does folate, although the donation of the methyl group by betaine is limited to the liver and the kidney. Although two meta-analyses did not find an overall association between folate intake and breast cancer risk (Lewis *et al*, 2006; Larsson *et al*, 2007), in some studies, folate intake was inversely associated with breast cancer risk in the whole population (Ericson *et al*, 2007), or among alcohol drinkers (Zhang *et al*, 1999; Rohan *et al*, 2000), or with certain hormone receptor types of breast cancer (Zhang *et al*, 2005; Larsson *et al*, 2008; Maruti *et al*, 2008). Choline and betaine may reduce the risk of breast cancer in a manner similar to that of folate. Despite the potential importance of choline and betaine in carcinogenesis, few epidemiological studies have evaluated the relationship between intakes of choline and betaine in relation to cancer risk. A case–control study found that choline intake was associated with a reduced risk of breast cancer (Xu *et al*, 2008). However, a prospective study did not find any associations between these nutrients and risk of pre-menopausal breast cancer (Cho *et al*, 2007a). To our knowledge, no prospective study has examined choline and betaine intakes in relation to breast cancer risk among post-menopausal women. We therefore examined intakes of these nutrients in relation to breast cancer risk in a large cohort of post-menopausal women.

## Materials and methods

### Study population

In 1976, we enrolled 121 700 female registered nurses aged 30–55 years in the Nurses’ Health Study (NHS) (Colditz and Hankinson, 2005). Biennial follow-up questionnaires were sent to the women to update information regarding diet and other lifestyle-related factors and to ascertain new diagnoses of major illnesses, including breast cancer. Deaths in the cohort were ascertained by reports from family members, from the postal service, and from a search of the National Death Index. We estimate that more than 98% of deaths were ascertained through these sources (Stampfer *et al*, 1984). The overall follow-up for this cohort is 90%. We considered women who reported that their menstrual periods ceased or underwent bilateral oophorectomy as post-menopausal. Women who reported menstrual cessation after undergoing a simple hysterectomy or removal of only one ovary or those who did not report the reason for cessation were considered post-menopausal when they reached the age at which natural menopause had occurred in 90% of the cohort (54 years for a current smoker and 56 years for a non-smoker). We limited the analysis to 37 374 women who were post-menopausal in 1984 and added otherwise eligible women as they became post-menopausal during follow-up. Thus, a total of 74 584 women were included in the analysis.

The procedures and protocols of the study were approved by the Institutional Review Boards of the Brigham and Women's Hospital.

### Dietary assessment

A semi-quantitative food-frequency questionnaire (FFQ) with ∼60 food items was sent to the members of the cohort in 1980. An expanded FFQ with ∼130 food items was administered to women in 1984, 1986, 1990, 1994, and 1998. Participants were asked how often, on an average, they consumed each type of food during the past year. Serving sizes were specified for each food in the FFQ. The questionnaire had nine possible responses, ranging from never or less than once per month to six or more times per day.

The choline and betaine composition of individual foods was added to the nutrient database of the FFQ (Harvard University Food Composition Database) using values published by Zeisel *et al* (2003a, 2003b) and from the choline database of the USDA (US Department of Agriculture, 2004). Betaine values were further updated in 2008 after correcting the betaine content of some common foods (breads and grains especially). The average daily intake of choline and betaine was calculated by multiplying the frequency of consumption of each food item by its choline and betaine content and summing the nutrient contributions of all foods. Total choline intake was calculated as the sum of choline intake from free choline, phosphocholine, glycerophosphocholine, phosphatidylcholine (lecithin), and sphingomyelin. We used the regression-residual method to adjust nutrient intakes for total energy intake (Willett and Stampfer, 1986). After examining the values of choline and betaine across different FFQs, we decided to start follow-up from 1984 because similar and more comprehensive FFQs were used since then, and thus intake data were more comparable across time.

As dietary intake may affect breast carcinogenesis over an extended period of time, to best represent long-term intake, we calculated cumulative averaged intakes of choline and betaine using repeated FFQ data for our primary analysis, to reduce measurement error in dietary assessment (Hu *et al*, 1999). For example, 1984 intake was used for the 1984–1986 follow-up period, and the average of 1984 and 1986 intake was used for the 1986–1990 follow-up to maintain a strictly prospective analysis.

Intakes of choline and betaine measured by our FFQ predicted plasma total homocysteine levels in a cohort study of men and women in the Framingham Offspring Study (Cho *et al*, 2006), which confirmed validity and biological relevance of choline and betaine intake measured by FFQ. For the lowest and highest quintiles of choline intake, the multivariate geometric means for homocysteine were 10.6 and 9.8 *μ*mol l^−1^ (*P* for trend <0.0001), respectively. For the lowest and highest quintiles of betaine intake, the corresponding geometric means were 10.4 and 10.1 *μ*mol l^−1^ (*P* for trend 0.05), respectively. The inverse association between dietary choline and betaine intake and levels of blood homocysteine was manifested primarily in participants with low folate intake (*P* for interaction <0.0001); among those with a folate intake of ⩽250 *μ*g per day, the geometric mean homocysteine concentrations in the lowest and highest quintiles of choline plus betaine intake were 12.4 and 10.2 *μ*mol l^−1^ (*P* for trend <0.0001), respectively. Although slightly weaker, a similar inverse association as in the Framingham Offspring Study was found between choline plus betaine intake and plasma homocysteine levels in the NHS. Compared with those in the lowest quintile, individuals in the highest quintile of choline plus betaine intake had 8% lower levels of homocysteine (Chiuve *et al*, 2007). Neither choline nor betaine intake individually was significantly associated with levels of homocysteine.

### Identification of cases

Biennial questionnaires mailed between 1984 and 2004 were used to identify newly diagnosed cases of breast cancer during follow-up. When a case of breast cancer was reported, we asked the participant (or next of kin for those who had died) for confirmation of the diagnosis and for permission to seek relevant hospital records and pathology reports. Only invasive breast cancer cases were included in the analysis. Confirmation of oestrogen receptor (ER) and progesterone receptor (PR) status was obtained by reviewing pathology reports.

### Statistical analysis

Participants contributed person-time from the date of return of the 1984 questionnaire until the date of breast cancer diagnosis, death, or June 2004, whichever came first. Participants were divided into quintiles according to their nutrient intakes. Relative risks (RRs) of breast cancer were calculated as the incidence rate for a given quintile of nutrient as compared with the rate for the lowest quintile. We used Cox proportional hazards regression to account for potential effects of other risk factors for breast cancer. To control as finely as possible for confounding by age, calendar time, and any possible two-way interactions between these two time scales, we stratified the analysis jointly on the basis of age in months at the start of follow-up and calendar year of the current questionnaire cycle. Multivariate models also adjusted simultaneously for smoking status, body mass index (BMI) at age 18 years, weight change between age 18 years and current cycle, height, physical activity, age at menarche, family history of breast cancer, history of benign breast disease, parity and age at first birth, post-menopausal hormone use, and intakes of calories, total folate, and alcohol. All covariates except BMI at age 18 years, height, age at menarche, and family history of breast cancer were updated in each questionnaire cycle or whenever new data were available.

We also examined the latency effect of choline and betaine intake using multiple dietary assessments. For example, for a latency period of 0–4 years, we used the 1984 intake for cases diagnosed from 1984 to 1986, the 1986 intake for cases diagnosed from 1986 to 1990, the 1990 intake for cases diagnosed from 1990 to 1994, and so on. For latency of 4–8 years, we used the 1984 intake for cases diagnosed from 1988 to 1990, the 1986 intake for cases diagnosed from 1990 to 1994, and so on. The 0–4-year latency analysis corresponds to the analysis using the most recent dietary intake. Latency analyses were used to examine whether there is a possible time lag between choline and betaine intake and risk of breast cancer.

The SAS PROC PHREG (Cary, NC, USA) tool was used for all analysis, and the Anderson–Gill data structure was used to handle time-varying covariates efficiently (i.e., a new data record was created for every questionnaire cycle at which a participant was at risk, with covariates set to their values at the time that the questionnaire was returned). For all RRs, 95% confidence intervals (CIs) were calculated. Tests for trend were conducted using the median value for each category of nutrient as a continuous variable. To test whether the association between choline intake and breast cancer risk was modified by alcohol intake, folate intake, BMI, post-menopausal hormone use, history of benign breast disease, and family history of breast cancer, cross-product terms for the level of an interaction variable (continuous form for folate and alcohol) and choline intake expressed as a continuous variable were included in the multivariate model. The *P*-value for the tests for interaction was obtained from a likelihood ratio test with two degrees of freedom. All *P*-values were two-sided.

## Results

During 532 338 person-years of follow-up of 74 584 post-menopausal women, we documented 3990 cases of invasive breast carcinoma. The age range of cases at the time of diagnosis of breast cancer was 38–82 years (mean=64, s.d.=7). Table 1 presents the distribution of risk factors for breast cancer by quintiles of choline and betaine intake in 1994, which is an approximate middle point of the follow-up. We have chosen the time point because participants could be added once they reach menopause after 1984, and thus the middle point of the follow-up may best represent the whole population in the analysis. Women with higher intake of choline were more likely to have higher BMI, higher physical activity, and higher folate intake (*P*<0.05). Women with higher intake of betaine were less likely to be smokers and more likely to have a history of benign breast disease, lower BMI, and higher folate intake (*P*<0.05). Choline intake in the NHS has reduced over time; the mean±s.d. values for choline intake were 338±82 mg per day in dietary assessment in 1984 and 298±69 mg per day in 2002, respectively. Betaine intake remained similar over time; the mean±s.d. values for betaine intake were 106±44 mg per day in dietary assessment in 1984 and 100±43 mg per day in 2002, respectively.

Higher intakes of dietary choline and betaine were not associated with reduced breast cancer risk (Table 2). Choline intake was associated with slightly elevated risk of breast cancer, although not statistically significant; the multivariate RRs (95% CI) for increasing quintiles of intake were 1.00 (referent), 1.09 (0.99–1.21), 1.10 (1.00–1.22), 1.09 (0.98–1.20), and 1.10 (0.99–1.22; *P*-value test for trend=0.14). Betaine intake was not associated with breast cancer risk. None of the individual sources of choline was related to breast cancer risk. Intake of methionine, another nutrient involved in one-carbon metabolism, was not associated with risk of breast cancer; the RR for top *vs* bottom quintiles of methionine intake was 1.08 (0.97–1.20). Intakes of choline and betaine were not associated with risk of breast cancer *in situ* (*n*=736; data not shown).

Results were similar when breast cancers were grouped by hormone receptor status. For example, the RRs for top *vs* bottom quintiles of choline intake were 1.07 (0.92–1.25) for ER+/PR+ cancers (*n*=1998), and 1.15 (0.86–1.54) for ER−/PR− cancers (*n*=538). As there may be a latency effect of choline and betaine, we took advantage of repeated assessment of dietary intake to assess the temporal relationship between choline and betaine intake and breast cancer risk. We evaluated latency periods of 0–4, 4–8, 8–12, 12–16, and 16–20 years. When the latency period became longer, the modest positive association between choline intake and breast cancer became weaker and in fact became slightly inverse for a latency period of 16–20 years (RR for the top *vs* bottom quintile of choline intake=0.92 (0.74–1.14)). There was no appreciable difference in association between betaine intake and breast cancer risk by latency period.

The availability of other dietary factors related to one-carbon metabolism, including alcohol and folate, may modify the association between choline and breast cancer risk. We thus examined choline intake and breast cancer risk by levels of folate intake (<300, 300–<400, 400–<500, and ⩾500 *μ*g per day) and alcohol intake (non-drinker, >0–<15, and ⩾15 g per day) (Table 3). The positive association between choline and breast cancer was limited to a mid-range folate intake of 300–<500 *μ*g per day (*P*-value for interaction=0.06). The association between choline intake and breast cancer risk did not differ appreciably by alcohol intake (*P*-value for interaction=0.31).

We also examined whether the association between intakes of choline and betaine and breast cancer risk differed by levels of other breast cancer risk factors, such as family history of breast cancer (yes, no), history of benign breast disease (yes, no), BMI (<27.5, ⩾27.5 kg m^−2^), and post-menopausal hormone use (never, past, or current) (Table 3). None of the *P*-values for interaction were statistically significant. However, choline intake was positively associated with breast cancer among those with no history of benign breast disease (RR for the top *vs* bottom quintile 1.25 (95% CI: 1.07–1.46); *P* for trend 0.03), but not among those with a history of benign breast disease (RR for the top *vs* bottom quintile 0.98 (95% CI: 0.84–1.13); *P* for trend 0.98). Similarly, choline intake was positively associated with breast cancer among current users of post-menopausal hormone (RR for the top *vs* bottom quintile 1.32 (95% CI: 1.03–1.70); *P* for trend 0.06), but not among those who never used hormone or who used hormone in the past (RR for the top *vs* bottom quintile 1.12 (95% CI: 0.94–1.33); *P* for trend 0.17 for never users and RR for the top *vs* bottom quintile 1.03 (95% CI: 0.88–1.21); *P* for trend 0.94 for past users).

## Discussion

In this large prospective study among post-menopausal women, we did not find any strong overall associations between intakes of choline and betaine and risk of breast cancer. The results were similar in breast cancer stratified by hormone receptor status and in the analyses stratified by several breast cancer risk factors.

Our findings on choline intake are not consistent with a case–control study of 1508 breast cancer cases that found that higher choline intake was associated with up to 24% lower risk of breast cancer, although the association was not linear (*P*-value for trend=0.29) (Xu *et al*, 2008). The study included both pre- and post-menopausal women and reported that the inverse association between choline intake and breast cancer risk was not altered by menopausal status. Betaine intake was not associated with breast cancer risk in the study, which was in accordance with our study. On the other hand, a large prospective study of pre-menopausal women did not find any associations between intakes of choline and betaine and breast cancer risk (Cho *et al*, 2007a). Few studies have examined choline intake in relation to other cancer end points (Cho *et al*, 2007b).

Choline and betaine are nutrients that were, until recently, not extensively investigated in epidemiological studies because of lack of food composition databases (Zeisel *et al*, 2003b). Choline and betaine are major dietary sources of methyl groups, as is folate. Choline is also a precursor of membrane phospholipids phosphatidylcholine and sphingomyelin. In animal studies, a choline-deficient diet itself can induce liver cancer in the absence of any carcinogen (Henning and Swendseid, 1996). Nutrients responsible for one-carbon metabolism, including folate and methionine, have been related to risk of cancer of several organs in humans (Giovannucci *et al*, 1993; Zhang *et al*, 1999; Alberg *et al*, 2000; Stolzenberg-Solomon *et al*, 2001; Martinez *et al*, 2004). Humans require choline from diet because of insufficient *de novo* synthesis in the body. The recommended daily intake was set in 1998 at 550 mg per day for men and 425 mg per day for women (Yates *et al*, 1998). Our previous data in this cohort showed that mean choline intake in this population of females was lower than the recommended daily intake (Cho *et al*, 2007b).

The positive association between choline intake and breast cancer risk, although modest and not significant, is similar to that which we found in relation to colorectal adenomas in this population; we found that higher choline intake was associated with up to 45% elevated risk of colorectal adenomas (Cho *et al*, 2007b). The latency analysis suggested that any positive association was mostly due to relatively recent choline intake, suggesting that choline may affect tumour progression, rather than tumour initiation. Biological explanations for the positive association may include the role of choline as a cell membrane phospholipid. Once a tumour is initiated, proliferation of tumour may in part depend on choline availability to generate cell membranes. In a study of hepatocarcinogenesis in rats, animals fed a choline-deficient diet for 3 or 6 months, followed by a choline-supplemented diet, had a higher incidence of tumours and more amplification of the c-myc oncogene than did animals fed a choline-deficient diet continuously, suggesting the role of choline in tumour development (Chandar and Lombardi, 1988; Chandar *et al*, 1989). Several cancers and cancer cell lines have altered membrane phospholipid metabolism with enhanced choline uptake and increased choline metabolite concentrations (Mori *et al*, 2004; Villa *et al*, 2005). Choline kinase, an enzyme converting choline to phosphocholine, which leads to the generation of membrane phospholipids, is elevated in human breast cancer (Ramirez de Molina *et al*, 2002).

The suggestive interaction between choline and folate intake in relation to breast cancer risk is hard to interpret, especially because the positive association between choline intake and breast cancer risk was mostly limited to middle-intake categories of folate. For colorectal adenomas, we found that the positive association between choline intake and adenoma risk was the strongest among those with the lowest folate intake (Cho *et al*, 2007b).

This study had several strengths. The prospective nature of the study avoided biases of case–control studies. Our study provided a unique opportunity to evaluate intakes of choline and betaine in a large population of post-menopausal women, which provided informative numbers of breast cancer cases in several stratified analyses. As we had repeated measures of dietary intake, we were able to examine long-term averaged diet, as well as several latency analyses. Measurement error is reduced by the use of repeated measures. Finally, we had information on a wide range of potential confounders and adjusted for them.

In conclusion, in this cohort of post-menopausal women, we found that intakes of choline and betaine were not related to a reduced risk of breast cancer.

## Figures and Tables

**Table 1:** Characteristics of the cohorts according to energy-adjusted choline and betaine intake among post-menopausal women in the Nurses’ Health Study in 1994^a^

	**Intake category (quintiles)**					
	**Choline**			**Betaine**		
**Variable**	**1**	**3**	**5**	**1**	**3**	**5**
Number of participants	10 195	10 216	10 222	10 191	10 245	10 212
						
*Group, %*						
Current smokers	13.4	12.6	13.9	17.7	13.0	9.8
History of benign breast disease	45.7	44.5	45.3	42.7	45.9	47.3
Family history of breast cancer	12.8	13.2	12.9	12.8	12.9	13.3
Current post-menopausal hormone use	41.4	44.3	43.5	40.2	44.4	45.7
						
*Mean*						
Age (years)	62.2	62.2	62.7	62.9	62.0	62.6
Height (inches)	64.3	64.4	64.5	64.4	64.4	64.4
Body mass index (kg m^−2^)	25.6	26.3	27.2	26.6	26.5	25.9
Physical activity (mets per week)	17.8	19.7	20.4	17.4	19.3	20.8
Age at menarche (years)	12.6	12.5	12.4	12.5	12.5	12.4
Age at first birth (years)	25.3	25.2	25.1	25.3	25.2	25.2
Parity (children)	3.1	3.1	3.1	3.1	3.1	3.1
Age at menopause (years)	47.6	47.7	47.4	47.4	47.6	47.6
Alcohol intake (g per day)	5.3	5.3	4.4	5.0	5.3	4.9
Folate intake (μg per day)	418.6	463.4	502.7	415.4	450.2	529.4

a Except for the data on mean age, all data shown are standardised to the age distributions of the cohorts in 1994. For simplicity, only data for the first, third, and fifth quintiles of intakes of choline and betaine are shown. All variables, except history of benign breast disease, family history of breast cancer, and parity for choline intake, and family history of breast cancer, height, age at first birth, and alcohol intake for betaine intake, showed a significant test for trend (*P*<0.05).

**Table 2:** Relative risk (RR) and 95% confidence interval (CI) of breast cancer (*n*=3990) according to quintile of cumulative averaged energy-adjusted choline and betaine intake in post-menopausal women

	**Quintile of intake**					
**Nutrient**	**1**	**2**	**3**	**4**	**5**	***P*-value, test for trend**
*Total choline*						
Median intake (intake range; mg per day)	260 (57–280)	294 (280–307)	319 (307–332)	346 (332–364)	396 (364–1427)	
No. of cases/person-years	721/208 365	821/208 457	825/208 667	816/208 847	807/208 723	
Age-adjusted RR (95% CI)	1.00	1.12 (1.01–1.24)	1.12 (1.01–1.24)	1.11 (1.00–1.22)	1.12 (1.01–1.23)	0.09
Multivariate^a^ RR (95% CI)	1.00	1.09 (0.99–1.21)	1.10 (1.00–1.22)	1.09 (0.98–1.20)	1.10 (0.99–1.22)	0.14
						
*Betaine*						
Median intake (intake range; mg per day)	71 (10–79)	86 (79–93)	99 (93–106)	114 (106–125)	144 (125–1595)	
No. of cases/person-years	764/208 378	786/208 206	798/208 644	858/208 549	784/209 282	
Age-adjusted RR (95% CI)	1.00	1.01 (0.91–1.12)	1.02 (0.92–1.13)	1.09 (0.98–1.20)	0.98 (0.89–1.09)	0.98
Multivariate^a^ RR (95% CI)	1.00	1.01 (0.91–1.11)	1.01 (0.91–1.12)	1.07 (0.97–1.19)	0.98 (0.89–1.09)	0.96
						
*Free choline*						
Median intake (intake range; mg per day)	58 (20–63)	66 (63–69)	72 (69–75)	79 (75–83)	90 (83–588)	
No. of cases/person-years	706/208 244	793/208 960	829/208 088	851/208 888	811/208 879	
Age-adjusted RR (95% CI)	1.00	1.09 (0.98–1.20)	1.11 (1.10–1.23)	1.14 (1.03–1.26)	1.10 (0.99–1.21)	0.07
Multivariate^a^ RR (95% CI)	1.00	1.07 (0.96–1.19)	1.10 (0.99–1.21)	1.12 (1.01–1.24)	1.08 (0.97–1.21)	0.13
						
*Choline from glycerophosphocholine*						
Median intake (intake range; mg per day)	33 (8–38)	42 (38–46)	50 (46–54)	59 (54–66)	77 (66–246)	
No. of cases/person-years	724/208 610	808/208 607	765/208 741	828/208 530	865/208 570	
Age-adjusted RR (95% CI)	1.00	1.07 (0.96–1.18)	1.00 (0.90–1.11)	1.06 (0.96–1.17)	1.09 (0.99–1.21)	0.11
Multivariate^a^ RR (95% CI)	1.00	1.06 (0.96–1.17)	1.00 (0.90–1.10)	1.05 (0.95–1.16)	1.09 (0.98–1.20)	0.15
						
*Choline from phosphocholine*						
Median intake (intake range; mg per day)	10 (0.3–11)	12 (11–13)	14 (13–15)	16 (15–18)	20 (18–92)	
No. of cases/person-years	761/208 510	759/208 406	810/208 561	805/208 734	855/208 848	
Age-adjusted RR (95% CI)	1.00	0.96 (0.87–1.06)	1.01 (0.92–1.12)	0.99 (0.89–1.09)	1.04 (0.94–1.15)	0.33
Multivariate^a^ RR (95% CI)	1.00	0.96 (0.86–1.06)	1.02 (0.92–1.13)	0.99 (0.89–1.10)	1.05 (0.95–1.17)	0.23
						
*Choline from phosphatidylcholine*						
Median intake (intake range; mg per day)	121 (18–133)	143 (133–152)	159 (152–168)	178 (168–192)	217 (192–967)	
No. of cases/person-years	795/208 353	820/208 581	817/208 598	770/208 898	788/208 630	
Age-adjusted RR (95% CI)	1.00	1.03 (0.93–1.13)	1.04 (0.94–1.14)	0.99 (0.90–1.09)	1.04 (0.94–1.15)	0.65
Multivariate^a^ RR (95% CI)	1.00	1.02 (0.92–1.12)	1.03 (0.93–1.14)	0.98 (0.89–1.09)	1.04 (0.93–1.15)	0.70
						
*Choline from sphingomyelin*						
Median intake (intake range; mg per day)	13 (0.01–14)	16 (15–17)	18 (17–19)	20 (19–21)	23 (21–90)	
No. of cases/person-years	751/208 238	843/208 411	797/208 578	827/208 955	772/208 877	
Age-adjusted RR (95% CI)	1.00	1.13 (1.02–1.24)	1.07 (0.97–1.18)	1.12 (1.01–1.24)	1.08 (0.97–1.19)	0.24
Multivariate^a^ RR (95% CI)	1.00	1.11 (1.00–1.22)	1.05 (0.95–1.16)	1.09 (0.99–1.21)	1.05 (0.95–1.17)	0.45

a Multivariate model was stratified by age in months at the start of follow-up and calendar year of the current questionnaire cycle and was simultaneously adjusted for smoking status (never, past, and current 1–14, 15–24, and 25 cigarettes per day), height (<63, 63–<64, 64–<66, 66+ inches), parity and age at first birth (nulliparous, parity ⩽2, and age at first birth <25 years, parity ⩽2 and age at first birth 25–<30 years, parity ⩽2 and age at first birth 30+ years, parity 3–4 and age at first birth <25 years, parity 3–4 and age at first birth 25–<30 years, parity 3–4 and age at first birth 30+ years, parity 5+ and age at first birth <25 years, parity 5+ and age at first birth 25+ years), body mass index at age 18 (continuous), weight change between age 18 and current (<−2, −2–2, 2.1–5, 5.1–10, 10.1–20, 20.1–25, ⩾25 kg), physical activity (quintiles of metabolic equivalents per week), age at menarche (<13, 13, ⩾14 years), family history of breast cancer (yes, no), history of benign breast disease (yes, no), use of post-menopausal hormones (never, past, current), and intakes of alcohol (continuous), energy (continuous), and folate (continuous).

**Table 3:** Multivariate relative risk (RR) and 95% confidence interval (CI) of breast cancer according to quintile of cumulative averaged energy-adjusted choline intake by breast cancer risk factors in post-menopausal women

	**Quintile of intake**						
**Breast cancer risk factor**	**1**	**2**	**3**	**4**	**5**	***P*-value, test for trend**	***P*-value, test for interaction**
*Alcohol*							
Non-drinker (*n*=936)	1.00	1.05 (0.85–1.29)	1.15 (0.94–1.42)	1.12 (0.90–1.38)	1.15 (0.93–1.41)	0.18	
<15 g per day (*n*=2106)	1.00	1.09 (0.95–1.26)	1.10 (0.96–1.27)	1.09 (0.95–1.26)	1.10 (0.95–1.28)	0.29	
15+ g per day (*n*=948)	1.00	1.22 (0.99–1.50)	1.09 (0.88–1.35)	1.08 (0.87–1.34)	1.10 (0.87–1.39)	0.80	0.31
							
*Folate*							
<300 *μ*g (*n*=1029)	1.00	0.95 (0.79–1.13)	1.01 (0.83–1.21)	0.99 (0.81–1.21)	1.04 (0.84–1.29)	0.69	
300–<400 *μ*g (*n*=943)	1.00	1.13 (0.91–1.39)	1.08 (0.87–1.34)	1.03 (0.82–1.28)	1.30 (1.04–1.62)	0.07	
400–<500 *μ*g (*n*=722)	1.00	1.39 (1.07–1.81)	1.28 (0.98–1.67)	1.47 (1.13–1.91)	1.39 (1.05–1.83)	0.04	
⩾500 *μ*g (*n*=1296)	1.00	1.02 (0.84–1.25)	1.12 (0.92–1.36)	1.01 (0.83–1.22)	0.94 (0.78–1.14)	0.29	0.06
							
*Post-menopausal hormone use*							
Never (*n*=1373)	1.00	1.01 (0.84–1.20)	1.08 (0.91–1.28)	1.06 (0.89–1.26)	1.12 (0.94–1.33)	0.17	
Past (*n*=1840)	1.00	1.09 (0.94–1.26)	1.05 (0.91–1.22)	1.02 (0.88–1.19)	1.03 (0.88–1.21)	0.94	
Current (*n*=777)	1.00	1.30 (1.02–1.66)	1.25 (0.98–1.60)	1.31 (1.03–1.67)	1.32 (1.03–1.70)	0.06	0.92
							
*History of benign breast disease*							
No (1877)	1.00	1.18 (1.01–1.37)	1.17 (1.00–1.36)	1.10 (0.95–1.29)	1.25 (1.07–1.46)	0.03	
Yes (2113)	1.00	1.01 (0.88–1.16)	1.05 (0.91–1.20)	1.08 (0.94–1.24)	0.98 (0.84–1.13)	0.98	0.24
							
*Body mass index*							
<27.5 kg m^−2^ (*n*=2574)	1.00	1.12 (0.99–1.26)	1.05 (0.93–1.19)	1.11 (0.98–1.25)	1.10 (0.97–1.26)	0.20	
⩾27.5 kg m^−2^ (*n*=1406)	1.00	1.02 (0.85–1.23)	1.13 (0.94–1.35)	0.97 (0.81–1.16)	0.98 (0.82–1.18)	0.55	0.15
							
*Family history of breast cancer*							
⩾No (*n*=3235)	1.00	1.14 (1.02–1.27)	1.09 (0.98–1.22)	1.09 (0.98–1.23)	1.09 (0.97–1.22)	0.36	
⩾Yes (*n*=755)	1.00	0.94 (0.73–1.19)	1.07 (0.85–1.36)	1.04 (0.82–1.33)	1.12 (0.87–1.44)	0.25	0.42

Multivariate model was adjusted for the same covariate as Table 2.
